# Treatment adherence redefined: a critical analysis of technotherapeutics

**DOI:** 10.1111/j.1440-1800.2012.00595.x

**Published:** 2013-03

**Authors:** Marilou Gagnon, Jean Daniel Jacob, Adrian Guta

**Affiliations:** a School of Nursing, Faculty of Health Sciences, University of Ottawa, Ottawa; b Dalla Lana School of Public Health, University of Toronto, Toronto, ON, Canada

**Keywords:** Foucault, HIV, power, surveillance, technology, treatment adherence

## Abstract

GAGNON M, JACOB JD and GUTA A. *Nursing Inquiry* 2013; **20**: 60–70 **Treatment adherence redefined: a critical analysis of technotherapeutics**

Treatment adherence issues in the context of chronic illnesses have become an important concern worldwide and a top priority in the field of health-care. The development of devices that will allow healthcare providers to track treatment adherence and monitor physiological parameters with exact precision raises important questions and concerns. The aim of this study is to interrogate the use of these new technological devices which allow for previously unavailable data to be recorded on an ongoing basis and transmitted via a tiny microchip inserted into the body. Drawing on the work of Michel Foucault, we analyze how this anatomo-political and bio-political instrument serves to discipline chronically ill individuals and govern the health of entire populations who suffer from chronic conditions. To support our analysis, this article comprises three sections. First, we provide an overview of treatment adherence and technotherapeutics. Then, we explain how technotherapeutics concern the government of bodies and conducts at the individual level and population level more generally. Lastly, we provide an example of how this analysis can be connected to routine nursing practice in the field of HIV.

My point is not that everything is bad, but that everything is dangerous. (Foucault 1984, 343)

In recent years, we have seen important advancements in the field of surveillance, thus witnessing a rise in the proliferation of its forms, a refinement in its technologies, and an expansion in/of its use (Yar 2003). These advancements are gradually changing the face of health-care and the way technology is being used to produce information about the bodily response to therapeutics. Health-care is, in fact, a domain where technologies are gradually moving inward to capture data not previously available and to generate new knowledge about patients. Pharmaceuticals have not, to our knowledge, been previously described as instruments of surveillance. However, recent developments suggest that they have the potential to monitor physiological responses and conducts (i.e., treatment adherence) with exact precision when combined with microchip technology. In an era where health-care is more technologically dominated than ever before, we argue that there is a need to critically examine the production of what we call *technotherapeutics* and their potential use within clinical settings. Furthermore, we posit that the development of technologically enhanced pharmaceuticals should be examined concurrently with the rise of *iiberveillance* (Michael and Michael 2010) – a new form of surveillance that will drastically change healthcare practices in the upcoming years.

According to Michael and Michael (2010), überveillance has to do with real time tracking and continuous monitoring whether it is of identity, time, location, physiological responses, or conducts. It is a type of surveillance that is always on and ever-present because it is embedded inside the human body via microchip technology (Michael and Michael 2010). It is the ultimate form of surveillance which allows information not previously available to be captured and transmitted to healthcare providers, probation officers, government authorities, and so on (Michael and Michael 2010). While this form of surveillance may seem promising for some, we consider that its application to the field of health-care raises numerous questions and concerns of a political and ethical nature. The aim of this paper is to interrogate the use of technotherapeutics for the continuous tracking and monitoring of treatment adherence in the context of chronic illnesses. Drawing on the seminal work of late French philosopher Michel Foucault, we argue that technotherapeutics serve to discipline chronically ill individuals and govern the health of entire populations who suffer from chronic conditions. Our critical (thus political) analysis draws on the Foucauldian concepts of governmentality and bio-power, which have been widely used to disrupt normalizing discourses in the field of health-care. To this end, we explore the ways in which technotherapeutics function as instruments of bio-power and problematize a mode of surveillance that is intrinsically linked to the logics of governmentality.

The growing inducement to develop and implement technotherapeutics may be examined through Foucault’s (2003a) use of *governmentality*, which describes the general mechanisms of society’s governance and does not refer specifically to how political units exercise authority and perform their routine functions. Rather, Foucault (1980, 39) uses governmentality as a conceptual framework to account for the ways through which capillary forms of power insert themselves into actions, attitudes, and discourses to produce a particular kind of individual and collective body – productive, but docile and easily governed. The impetus to act on populations in this way creates a cycle that further necessitates the development of governmental apparatuses and knowledge that ‘allow for the exercise of this very specific albeit complex form of power’ (Foucault 2003a, 244). As such, Foucault (1990) notes a continual definition and redefinition of tactics of governmentality over time – all of which are concerned with the management of individuals as members of a *population*. The development and implementation of technotherapeutics are suggestive of particular tactics of governmentality that concern issues of individual conduct (i.e., treatment adherence), which are also known to interfere with the production of a healthy population.

Expanding on the concept of governmentality, Foucault (1990, 140) introduces *bio-power*– with the two poles of *anatomo-politics and bio-politics*– to account for the historical ‘explosion of numerous and diverse techniques for achieving the subjugation of bodies and the control of populations’. At one end, is described the anatomo-political pole which is ‘centred on the body as a machine: its disciplining, the optimization of its capacities, the extortion of its forces, the parallel increase in its usefulness and its docility, [and] its integration into systems of efficient and economic controls’ (Foucault 1990, 139). At the other end, is described the bio-political pole which is focused on supervision of ‘the species body, the body imbued with the mechanics of life and serving as the basis of the biological processes: propagations, births and mortality, the level of health, life expectancy and longevity, with all the conditions that can cause these to vary’ (Foucault 1990, 139). These two poles – the discipline of the body and the regulation of the population – are central to an analysis of the organization of life (Foucault 1990). By considering both poles in our analysis, we deviate from the conventions of traditional healthcare analysis – focused on the ‘problem’ of adherence – and turn instead toward the politics of adherence work; thus locating the problem of adherence in a larger social apparatus. We will revisit and further elaborate on these two poles in our subsequent analysis of technotherapeutics.

## Background

Treatment adherence issues in the context of chronic illnesses have become a major concern worldwide and a top priority in the field of health-care. Indeed, treatment adherence rates – the extent to which a patient takes a treatment as prescribed and follows the recommendations of the prescriber (lifestyle, diet, etc.) – are about 50% in the general population (Haynes et al. 2008). According to the World Health Organization (2003), treatment adherence rates are particularly low in individuals who suffer from a chronic condition and must undergo prolonged treatments. In fact, the extent to which chronically ill individuals take their treatment as prescribed and follow the recommendations of the prescriber will often decrease over time, thus leading to undesirable health outcomes (World Health Organization 2003). Research suggests that low treatment adherence rates in this particular population may lead to poor clinical outcomes (i.e., symptom management), less than optimal disease management (i.e., disease progression), and additional health-care costs (Ostenberg and Blaschke 2005). In the United States alone, the annual costs related to poor treatment adherence are estimated at 290 billion (Beni 2011). Underpinned by a growing understanding that treatment non-adherence is a major threat to the health and wealth of countries worldwide, many stakeholders have expressed the need to develop effective interventions and innovative tools to maximize adherence rates among people who suffer from chronic conditions (Haynes et al. 2008).

Interventions to address non-adherence and to maximize treatment uptake among individuals who suffer from chronic conditions are multiple. They not only emerge from the need to ensure clinical outcomes and optimal disease management within this population, but from an imperative of cost containment. Much of what is being developed in the field of adherence research is focused on the need to monitor adherence and increase interactions with individuals who undergo prolonged treatments. As such, monitoring methods (direct or indirect) provide information of utmost importance for healthcare providers and are considered particularly useful to optimize treatment adherence (Haynes et al. 2008). Direct methods may include direct observation by healthcare providers (i.e., pharmacist) or objective measurements of drug concentrations in blood or urine. Thus, these methods not only require the presence of a healthcare provider who will record treatment adherence, but imply that individuals who deviate from the prescribed treatment will be automatically identified. In this sense, direct methods allow for healthcare providers to keep track of adherence via objective measurements (i.e., number of visits at the pharmacy, number of pills taken, and serum concentration) and intervene directly when individuals fail to take their treatment as prescribed.

Indirect methods, on the other hand, rely on information provided by the individual under treatment (i.e., number of missed doses) and the subjective assessment of overall adherence rate by a healthcare provider. In this case, adherence rate may be estimated through interviews, pill counts, frequency of prescription refills, and so on. These methods, however, fail to provide an accurate measurement of treatment adherence and precise information about the person under treatment. Recent advancements in technology now enable healthcare providers (and researchers) to monitor adherence indirectly via an electronic system capable of recording, for example, when medication bottles are opened (smart pill bottles) or when pumps are activated. Indirect methods such as this one have created new possibilities for healthcare providers to objectively monitor adherence at a distance and intervene directly when individuals fail to take their treatment as prescribed. Despite obvious difficulties in providing a fine measurement of treatment adherence, which is based solely on the opening of a bottle or the activation of a pump, this innovative (and indirect) method has opened the doors to new ways of monitoring adherence in individuals who suffer from chronic conditions and ensuring that those who deviate from the prescribed treatment are identified in a timely fashion.

Recently, the field of adherence research has taken a dramatic step forward by developing devices that will allow for healthcare providers to track adherence and document physiological parameters with exact precision. In March of 2010, the University of Florida announced that its engineering researchers had successfully added a tiny microchip and digestible antenna to a standard pill capsule (Hoover and Howell 2010). Once ingested, the pill communicates with a stand-alone electronic device carried or worn by the patient. Next, the device signals a cell phone or laptop to confirm that the pill has been ingested by the patient. The private sector has also engaged in the development of a similar product and has announced that it will soon market ‘intelligent pharmaceuticals’. Proteus Biomedical^1^ is currently collaborating with pharmaceutical companies in the development of these so-called intelligent pharmaceuticals with the objective of transforming the clinical management of chronic conditions. As such, they have developed an ingestible technology that works in a two-step process as well as an implantable microchip that is designed to transmit information to healthcare providers. By adding a digestible sensor to a standard pill capsule, the sensor undergoes an activation process within the stomach fluids and sends digital signals to the implantable microchip located under the skin of individuals who undergo prolonged treatments. For Proteus Biomedical, this device offers significant advantages for healthcare providers because it is capable of tracking the date and time of pill ingestion, recording drug-related information (i.e., type, dose, and place of manufacture), and measuring physiological parameters (heart rate, blood pressure, weight, blood glucose, body temperature, and respiratory rate). This prototype is set to record information, generate feedback in real time for those involved in adherence work (including healthcare providers, patients, family members, and relatives), and promptly signal when a treatment is not being taken as prescribed.

These new technologies are being developed and promoted under the premise that they can improve the therapeutic management of chronic conditions, maximize clinical outcomes, facilitate communication with healthcare providers, and individualize the care provided to those who undergo prolonged treatments. We argue, however, that technotherapeutics will be deployed to discipline chronically ill individuals by means of continuous surveillance and govern the health of entire populations who are seen as non-compliant, potentially dangerous, and overly costly. In the following segment, we will expand on the concept of bio-power and how it relates to the introduction of technotherapeutics in the clinical management of chronically ill individuals. We will examine how this instrument of surveillance is, in fact, an anatomo-political instrument that exerts a hold over individual bodies and reconfigures individual behaviors in accordance with a pre-determined set of clinical objectives. We will also examine how technotherapeutics make possible the regulatory control of populations and the calculated management of health itself. This, according to Foucault (1990), is tied to economic and political imperatives central to bio-power.

## Continuous Surveillance: On the Importance of Disciplinary Power

From a Foucauldian perspective, anatomo-politics encompasses a range of technologies capable of producing docile bodies and training individuals to operate in particular ways (Foucault 1995). In *Discipline and Punish*, Foucault (1995) argues that these technologies are productive in the sense that they operate to produce disciplined individuals. That is, they produce effects on the conducts, habits, and attitudes of these individuals who are encouraged to take part in their own regulation and achieve pre-determined objectives (i.e., clinical objectives). Discipline, here, is achieved through a subtle yet transformative form of power – known as *disciplinary power*– and the use of rather simple technologies. To better understand the success of disciplinary power, one must take a closer look at the technologies used to produce docile bodies and train individuals in line with specific objectives.

Foucault (1995) identified three simple technologies to illustrate how disciplinary power functions: hierarchical observation, normalizing judgment and examination. For the purpose of this analysis, we draw our attention to the hierarchical observation and the role of surveillance in the disciplining process. The *panopticon* or *panopticism* is an important concept that Foucault (1995) used to theorize surveillance and disciplinary power. Based on Jeremy Bentham’s work on prisons in 18th century Britain, the panopticon (Greek for everything, place of sight, or all-seeing) represented an idealistic architectural design whose optic effects permitted continuous surveillance and control over its captive population (Ellin 1997). Composed of a central observation tower encircled by an annular building where individual cells are positioned, the panopticon enabled a prison guard to see every inmate from a central viewpoint, while at the same time preventing the inmates from seeing who is watching (Holmes 2001). Under the guise of complete visibility, explains Foucault (1995), lie the disciplinary function of surveillance and its subsequent power over individuals who are rendered exposed within the panoptic machine. Thus, individuals who are held captive within the panoptic machine come to be disciplined simply by their exposure to the gaze of a watcher (Foucault 1995). Living in a state of conscious and *permanent visibility* (Foucault 1995), these individuals experience firsthand the effects of disciplinary power and eventually come to self-discipline. The success of this disciplinary technology can be explained by the fact that the ones being observed know that they are observed and, as a result, end up internalizing such surveillance (Foucault 1995). In this sense, the panopticon represents an ideal structure where individuals can be transformed, trained, disciplined, and normalized at a distance – without recourse to physical or verbal contact (Foucault 1995).

It is important to highlight that disciplinary power is most effective when hierarchical observation and the knowledge it produces are combined with normalizing judgment and examination. In other words, discipline functions best when the information obtained by means of surveillance is incorporated in a system of micro-penalties, a system in which ranking serves as punishment or reward (Foucault 1995). What prevails in the work of Foucault (1995) is that all disciplinary regimes include corrective strategies (micro-penalties) that encourage the adoption of prescribed conducts, habits, and attitudes. This particular instrument is often used in combination with examination procedures through which it becomes possible to qualify and to classify the performance of individuals according to a set of pre-determined objectives (Foucault 1995). The intent is to be able to sanction the weak while validating those performances that meet expectations based on the knowledge gathered through examination procedures (Foucault 1995). At this point in the paper, it is interesting to note that disciplinary technologies are not restricted to carceral institutions and can, in fact, be employed outside of this institutional frame. Today, these technologies can take many forms and are apparent in a complex web of technologically enhanced surveillance tools. In an era when new surveillance tools are expanding the medical gaze outside the walls of the clinic and allowing for treatment adherence to be monitored on an ongoing basis, Foucault’s work is of utmost importance. We consider that his meticulous description of disciplinary power allows us to address some of the questions, concerns, and implications not yet discussed in the literature. The next section explores some of these questions, concerns, and implications in more detail.

## Treatment Adherence as Discipline: Domesticating Chronically Ill Individuals

Of particular importance to this discussion is the relationship between new surveillance mechanisms, adherence work, and the disciplinarization of chronically ill individuals. By adherence work, we mean the broad range of activities through which healthcare providers, family members, relatives, and patients themselves look after treatment uptake to achieve optimal clinical outcomes. What becomes evident is that the need to closely monitor treatment adherence, and ensure those who deviate from the prescribed treatment are identified in a timely fashion, has led to the development of a new panoptic machine. While the architectural design of this panoptic machine cannot be seen to the naked eye, it follows the imperatives envisioned by Bentham and described by Foucault (1995): at the periphery, a virtual network; at the center, a computer is open onto a multiscreen interface; the virtual network is divided into eFiles, each of which corresponds to a specific patient; they have two separate channels, one that receives signals from a number of implantable devices (each of which is assigned to a specific patient); the other that logs information into a neatly organized database. All that is needed to make this design work is for patients to understand that they are being watched and unrelentingly exposed to an immaterial gaze. The insistent pressure of this immaterial and rather asymmetrical gaze is essential to the functioning of the panoptic machine (Foucault 1995) and the disciplinarization of the individuals who are under watch within this particular structure. Here, the exercise of discipline presupposes a structure that governs by means of surveillance – a structure where it is possible to induce a state of *permanent visibility* (Foucault 1995).

The development of technotherapeutics to track treatment adherence and monitor physiological parameters proves to be an extension of surveillance in its most penetrating ways. Continuous electronic surveillance using these new technologies allows information that was not previously available to be captured within the body and transmitted directly to healthcare providers. Evidently, this new form of surveillance is highly effective. The surveillance apparatus could be temporarily shut down and yet the effects would remain the same because chronically ill individuals would continue to believe that they are under watch. Writing from a Foucauldian perspective, Holmes (2001, 9) considers that this phenomenon can be explained by the fact that ‘the trap of visibility closes in on those who believe they are always observed, and ends by the observed internalizing the surveillance and unwittingly taking the task and observing themselves’. Here, it is important to recognize that the efficiency of the panoptic machine can be explained by the fact that surveillance not only makes individuals aware that they are being watched, but it makes them engage in self-surveillance during times of deviance; or, before misconducts or faults (such as non-adherence) even take place (Holmes 2001). Unsurprisingly, the intensification of surveillance is considered by some to be the best strategy to address treatment non-adherence and render individuals more efficient in the management of their chronic health conditions. This strategy relies on the fact that chronically ill individuals will experience firsthand the effects of continuous surveillance and, as a response, make optimal use of their capacities in following the recommendations of the prescriber. As such, the introduction of technotherapeutics to the field of treatment adherence makes it possible to produce docile bodies and train individuals to operate in line with clinical objectives.

Disciplinary power functions through hierarchical observation, as discussed earlier, but is more productive when combined with normalizing judgment and examination. What technotherapeutics allow healthcare providers to do is to produce new knowledge about chronically ill individuals and use this knowledge to qualify and to classify their performance according to a set of pre-determined clinical objectives. The idea here is to use this new and previously unavailable knowledge to sanction individuals who demonstrate poor adherence while validating those performances that meet expectations. From this perspective, it is believed that individuals will be motivated to adopt prescribed conducts, habits, and attitudes when they are confronted with their performance (optimal or not) and positioned in relation to the norm. The primary and desired effects sought by this normalizing process are to encourage self-discipline and nourish the desire of each individual to conform to expectations. This process is fundamental to the functioning of disciplinary power; hence, pre-determined adherence rates that serve to constitute the norm not only encourage desired conducts, habits, and attitudes but allow for an intervention to take place in times of deviance (non-adherence). Technotherapeutics make it possible to intervene promptly in this particular situation and leave very little maneuverability for chronically ill individuals who must cope with the many challenges they face on a daily basis.

## Bio-Politics and the Management of Chronically Ill Populations

If anatomo-politics works to discipline the individual, then bio-politics seeks to regulate the life processes of entire populations ‘so as to optimize a state of life’ (Foucault 2003b, 246). Bio-politics operates as the state (and its institutions) becomes more knowledgeable about specific populations and more involved in their regulation – including the regulation of life processes such as birth, death, health, propagation of diseases, sickness, sexuality, and so on (Foucault 1990). Bio-politics, explains Foucault (1990), is closely tied to surveillance and the production of knowledge about populations. In fact, the birth of bio-politics is said to coincide with the introduction of new techniques to study and closely monitor biological occurrences at the population level (Foucault 1990). Lemke (2011) explains that the emergence of statistics, demography, epidemiology, and biology have made it possible to study life processes as new objects of political reflection and produce detailed knowledge about populations (and individuals as members of those populations). It has also contributed to the development of bio-political interventions to optimize life processes (as new objects of political action) and ensure more effective regulation at the population level (Lemke 2011). Here, it is important to understand that the detailed knowledge produced by statistical, demographic, epidemiological, and biological analyses constitute the very basis of, and justification for, bio-political interventions (Foucault 1990). Thus, the linkage between the production of knowledge (by means of surveys, census, epidemiological studies, new technologies, and so forth) and the potential for more effective regulation is one that cannot be understated.

Bio-politics stands for the administration of life as a collective reality (Lemke 2011) and the management of issues known to interfere with life processes. It is concerned with issues that can be documented, measured, and aggregated on the level of populations – but, also, with calculations of possible and probable risks (Gordon 1991). To this end, bio-political interventions take on the semblance of solutions to discrepancies uncovered in the process of gathering information about populations or in the process of calculating risks within the collective body. The expansion of politics to the calculation and the administration of risks have given rise to a number of interventions for ‘reducing the probability of untoward events across the population’ (Rose 2007, 71). According to Rose (2007, 70), this shift is compatible with modern bio-politics and the deployment of ‘a variety of strategies that try to identify, treat, manage, or administer those individuals, groups, or localities where risk is seen to be high’. This shift has necessitated the expansion of the medical jurisdiction and authority ‘to the management of chronic illness and death, the administration of reproduction, the assessment and government of “risk”, and the maintenance and optimization of the healthy body’ (Rose 2007, 10). The old techniques of medicine, what could be seen and known, have been advanced through the rise of biotechnologies which harness the information and life sciences to ‘see’ and ‘do’ more than has ever been possible (Rifkin 1998). As suggested by Rose (2007), medicine has been transformed into *technomedicine*, and healthcare institutions now function as places of surveillance and knowledge production highly preoccupied with and involved in the regulation of health.

Much has been written about the political economy of health in neoliberal societies (Coburn 2000; Armstrong, Armstrong, and Coburn 2001; Harvey 2005; Navarro 2007) – societies where patients are constructed as entrepreneurial subjects gifted with freedom, autonomy and the capacity to properly care for themselves and others (Rose 1996; Petersen 1997). From this perspective, health is taken up and mobilized through what Murray (2009) describes as the interrelated processes of bioeconomics, biomedicalization, and biocultural discourses. Here, the notion of bioeconomics helps us understand the business of health in which neoliberal political economic policies and biomedical, pharmaceutical, and state discourses intertwine, affirming their respective dominance, and demanding greater efficiency and more effective regulation (Murray 2009). In recent years, the patterns associated with bio-politics have extended beyond individual state responses and have become evident in attempts to manage and regulate health on a global level through institutions such as the World Health Organization, United Nations, and philanthropic non-governmental organizations (Bashford 2006; Brown and Bell 2008; Brown and Watson 2009). These institutions play an important role in the optimization of health and the government of risks globally. They also contribute to the deployment of health optimization products and the development of policies and programs which are largely based on the idea that individuals as members of a global collective must act as informed entrepreneurs by consuming the best resources available to preserve and maintain their human capital (Petersen 1997). As such, we consider that the notions of bio-politics, neoliberalism and bioeconomics are of utmost importance to this analysis. These notions allow us to critically examine the tensions at play in the development of technotherapeutics and the intensification of efforts to govern chronic illness. In the following segment, we explore some of these tensions and expand on the use of technotherapeutics as an instrument of bio-politics.

## Governing Chronic Illness: Technotherapeutics as an Instrument of Bio-Politics

Foucault (1990, 139) explains that the shift to bio-political regulation does not constitute the disappearance of disciplinary techniques, but that regulation works simultaneously and in parallel with the techniques of discipline. We locate the development and growth of technotherapeutics at the intersections of bio-politics, and what Clarke et al. (2003) have termed *biomedicalization*. Biomedicalization, they argue, extends the process of medicalization and:

…is characterized by its greater organizational and institutional reach through the meso-level innovations made possible by computer and information sciences in clinical and scientific settings, including computer-based research and record-keeping. The scope of bio-medicalization processes is thus much broader, and includes conceptual and clinical expansions through the commodification of health, the elaboration of risk and surveillance, and innovative clinical applications of drugs, diagnostic tests, and treatment procedures (Clarke et al. 2003, 165).

In what they further term *biomedical governmentality*, Clarke et al. (2003, 174) argue ‘computerization allows more aspects of life to be scrutinized, quantified, and analyzed for their relationships to health and disease’. The development of technotherapeutics brings technology and the body into a regulatory framework that was only previously imagined in science fiction. Bryan (2009, 87) has eloquently stated that such ‘biotechnology hails us in terms of bio-politics. It promises to remedy deficiencies long thought endemic to the human condition, and now hails them as politically manageable’.

In the context of chronic illnesses, healthcare providers operate at the level of chronically ill individuals concurrently with that of chronically ill populations to supervise adherence to prescribed treatments and pay particular attention to those who are not engaging in ‘healthy’ practices. They also convey information about treatment adherence and intervene through various forms of counseling to make certain chronically ill individuals actively participate in their own regulation. However, any interest in these individual’s actions is important only to the extent that they become politically useful and productive (Dreyfus and Rabinow 1982). The goal is to identify, prevent, contain, manage potential issues with prescribed treatments, and gather information about targeted populations who receive care. Technotherapeutics brings together three major forms of risk identified in neoliberal societies; insurance risk, epidemiological risk, and case management (Dean 2010). These three conceptions of risk are less concerned with individual adherence than with managing shared health costs, the spread of disease, and keeping individuals who pose a risk connected to regulatory systems. Risk assessment is no longer an individual matter, but now accounts for whole groups and their practices, and the social risk they pose in relation to health and disease, life and death.

A Foucauldian analysis recognizes ‘the work that individuals perform upon themselves in order to become certain kinds of subjects’ (Hamann 2009, 38). This is especially true when the awareness and self-management of one’s health has become a moral technology (Lupton 1999). We understand that technotherapeutics (as many other health technologies) may be initially welcomed by many of those they are intended to help. These technologies may provide individuals who are chronically ill with a sense of identity and even ‘empowerment’ about their adherence. However, those subjected to these biotechnologies may come to think of themselves as constituted through the ‘facts’ produced (the individual and aggregate outputs) and the biotechnological discourses which surround their deployment (Robertson 2001; Bryan 2009). As has been observed historically, these technologies may ultimately become divisive and serve to differentiate the ‘good’ from the ‘bad’, or, in a bio-political sense, to differentiate those worthy of life (ongoing treatment and support) and those who the state should ‘let die’ (denied future medication or insurance coverage). Overall, we understand technotherapeutics as serving to both discipline individual bodies and also to regulate whole groups of people deemed to constitute a threat to the collective body. In this sense, we consider that adherence work is above all a political project that endeavors to achieve optimal disease management (through surveillance and discipline), reduce the financial burden of treatment non-adherence on healthcare systems, and serve to further marginalize and differentiate ‘at-risk groups’ because of their unwillingness or inability to conform.

## Treatment Adherence in the Field of HIV

To further illustrate our argument, we will examine more closely the example of HIV. The management of HIV infection has necessitated dramatic governmental responses through various focused medical, legal, social, and political interventions at the state and intra-state level. Within an orientation to the productive value of the population, people living with HIV are understood as ‘risky’ and deemed as being in particular need of discipline and regulation. We have chosen this particular case because of our own practice experience and concerns we share about how this group of chronically ill individuals will be negatively affected by technotherapeutics. We do not mean to suggest that people living with HIV represent a homogenous group, recognizing the diversity of individuals and communities affected by HIV, or that only people living with HIV will be affected. Rather, we anticipate that the use of these technologies may first become normalized within this group (where the greatest ‘need’ is seen) and then become normalized and spread outwards.

The clinical management of HIV infection revolves around a complex assemblage of potent molecules that inhibit viral replication, commonly referred to as highly active antiretroviral therapy (HAART). The introduction of HAART in 1996 has radically transformed the prognosis of people living with HIV by producing a dramatic decrease in morbidity and mortality rates associated with the progression of HIV infection. To ensure treatment efficacy and prevent the development of viral mutations, which can lead to treatment resistance, people living with HIV must demonstrate exceptional adherence on a daily basis. Research indicates that consistently high levels of adherence are necessary to achieve optimal viral suppression and prevent treatment resistance (Paterson et al. 2000). Research also indicates that high levels of adherence are necessary to suppress viral load over time and decrease the risk of HIV transmission (Lima et al. 2007). Yet treatment non-adherence has been shown to range from 33% to 88% in people living with HIV (Mill et al. 2006). This leads us to think that people living with HIV may be seen as risky and in need of more effective regulation. Certainly, then, technotherapeutics could take on the semblance of a solution to discrepancies uncovered in the process of gathering information on treatment adherence within this particular population and calculating the risks of non-adherence.

In recent years, there has been a call for interventions to improve and maintain adherence rates among people living with HIV. In the clinical area, people living with HIV are constantly reminded of the importance of treatment adherence and the need to reconfigure their behaviors to achieve a pre-determined set of clinical objectives. They are encouraged to strive for an undetectable viral load, which is indicative of optimal adherence and considered necessary for preventing treatment resistance, treatment failure, HIV transmission, disease progression, and possibly death. Those who fail to demonstrate high levels of adherence are targeted by healthcare providers and must undergo various forms of adherence counseling generally performed by nurses. Yet, this ‘failure’ to adhere is more complicated than is often characterized. Adherence is affected by a range of issues, including physical and cognitive functioning, treatment complexity and adverse effects, stigma and fear of disclosure, and competing pressures resulting from mental health and addictions issues (Mehta, Moore, and Graham 1997; Duran et al. 2001; Schambelan et al. 2002; McArthur, Brew, and Nath 2005; Rintamaki et al. 2006; Sonneville et al. 2011). It is also mediated by a number of variables as basic as the availability of transportation (to get medication) and food (to take it with) (Hardon et al. 2007).

The potential use of technotherapeutics in the clinical management of people living with HIV is particularly concerning because it ties perfectly well with recent efforts to intensify surveillance and treatment as means of prevention. Popularly termed ‘treatment as prevention’, this concerted response purports to reduce HIV transmission by lowering the viral load of people living with HIV through early initiation of treatment (Granich et al. 2009; Check Hayden 2010; Granich et al. 2010; Conway and Tossonian 2011; Taege 2011). ‘Treatment as prevention’ initiatives have taken a particular focus on ‘hard to reach’ and ‘vulnerable populations’ through a ‘seek and treat’ approach (Johnston et al. 2010). Returning to the bio-political goal of ‘making live’ and ‘letting die’, we understand that the populations deemed ‘hard to reach’ and ‘vulnerable’– gay and other men who have sex with men, sex workers, injection drug users, and aboriginal people – have been historically constructed as ‘risky’ and dangerous. In this particular context, technotherapeutics would allow for healthcare providers to gather previously unavailable information about these populations and use this information to intervene directly with patients who deviate from prescribed treatments; not for their benefit, but to make sure they do not affect those who the state would ‘make live’ (otherwise useful and productive bodies).

This new form of surveillance would be particularly useful in situations where individuals are seen as non-compliant and even ‘dangerous’. Although outside the scope of our analysis, commentators have already raised concerns about the possibility of criminalizing treatment refusal within a framework of treatment as prevention (Straub 2011). Technotherapeutics would allow healthcare providers to identify those who ‘fail’ to maintain an undetectable viral load because of poor treatment adherence and generate information that may very well be used to sanction these patients within the medical domain but also beyond with legal implications. In this sense, technotherapeutics would not only reinforce surveillance but also create new opportunities for the management of people living with HIV. Patton (2011) has raised concerns about the use of treatment as prevention and the intensification of efforts to make people living with HIV less infectious, which fail to consider the realities of living with HIV and managing HAART on a daily basis. There remain ongoing issues with the treatment offered to people living with HIV and unresolved problems with treatment coverage that cannot be ignored. We are compelled to ask how technotherapeutics would benefit people living with HIV and call for caution to be exercised over their implementation and use in clinical settings.

## Final Remarks

In this paper, we have argued that the introduction of technotherapeutics gives rise to a new form of surveillance (i.e., überveillance) whereby chronically ill individuals are always under watch and constantly monitored for treatment adherence. We examined the ways technotherapeutics serve to discipline chronically ill individuals and govern the health of entire populations who suffer from chronic conditions. In light of our analysis, we consider that the development of these new technologies and their use within the clinical setting require more reflection. On one hand, technotherapeutics are being introduced under the premise that they can improve the therapeutic management of chronic conditions, maximize clinical outcomes, facilitate communication with healthcare providers, and individualize the care provided to those who undergo prolonged treatments. On the other hand, the development and implementation of technotherapeutics suggest particular tactics of governmentality that cannot be overlooked. In conclusion, we consider that adherence enhancing technologies such as the ones described in this study fail to highlight neglected politics surrounding their application. Somehow, our concern with treatment adherence has promoted a shift in emphasis away from what transpires in the clinic toward what is happening outside of it. We see here evidence that überveillance may provide more sophisticated ways of governing chronic illness at a distance and make them constant and embedded (Michael and Michael 2010). Although this new form of surveillance may improve clinical management of chronically ill individuals from a biomedical perspective, it remains to be seen, at what cost. We hope that this analysis will encourage further discussions around the potential use and implications of technotherapeutics in the context of chronic illnesses. Undoubtedly, the need exists for more discussions of political, ethical, and professional nature.

## Footnotes

1 For more information, see: http://www.proteusbiomed.com/.
